# New Appliances and Things Medical

**Published:** 1895-06-01

**Authors:** 


### NEW APPLIANCES AND THINGS MEDICAL.

### AUSTRALIAN WINES, "EAGLE BRAND."

(R. H. Gate and Co., 28, Gracechurch Street, E.C.)

The value of Australian wines for medical purposes is now so generally accepted that we are glad to have the opportunity of noting that the proprietors of the Eagle brand are in a position, through their extensive stock of matured wine, to place on the English market wines of uniform quality. The samples sent to us for examination consisted of representative kinds of Australian Burgundy, Chablis, and claret, and our analyst has made a separate report on each, which we have pleasure in reproducing in full, as it confirms the statements made by the proprietors that these wines are all of unblended vintage, and represent the pure juice of the grape. The samples analysed were: (1) Chateau St. Henri, Burgundy, described as of great body and fineness, equal to the higher qualities of the Côte d'Or; (2) Chateau St. Henri, Chablis, of great vinosity; (3) Chateau St. Henri, claret, the product of the vine transplanted from the best districts of the Margaux; full bodied, with delicate flavour and aroma, capable of great improvement by age in bottle.

Analysis in Parts per 100.

| | Burgundy. | Chablis. | Claret. |
|---|---|---|---|
| Alcohol by weight ... | 9·86 | 11·0 | 9·79 |
| Extractive matters ... | 2·27 | 1·87 | 2 01 |
| Mineral mattter ... | 0·23 | 0·2 | 0·18 |
| Total acidity (N/10 acid per 100 c.c.) ... ... | 97·6 | 63·2 | 93·6 |
| Volatile acidity (N/10 acid per 100 c.c.) ... | 42·8 | 20·8 | 41·2 |

These acidities, when calculated to tartaric acid and acetic acid respectively, give the following quantities per 100 parts by volume:—

| | Burgundy. | Chablis. | Claret. |
|---|---|---|---|
| Acetic acid ... ... | 0·257 | 0·125 | 0·247 |
| Tartaric acid ... | 0·411 | 0·318 | 0·393 |

The acidity of the Burgundy and claret so far as tartaric acid is concerned is similar to that obtained in equivalent European wines, but the volatile acid is somewhat higher. The quantity of glycerine present in the three wines has also been determined, and from its ratio to that of the alcohol found there is evidence that there has been no addition of alcohol to the wine after fermentation. The proprietors guarantee that all their wines are similarly free from added spirit. The bouquet and taste of the samples we have examined are satisfactory. The wines are of superior quality, and we can cordially commend them to all who desire to obtain Burgundy, Chablis, or claret in excellent condition. The samples submitted may be described as good sound wines.

